# Causal association of sleep disturbances and low back pain: A bidirectional two-sample Mendelian randomization study

**DOI:** 10.3389/fnins.2022.1074605

**Published:** 2022-12-02

**Authors:** Ge Luo, Yuanyuan Yao, Jiachun Tao, Tingting Wang, Min Yan

**Affiliations:** Department of Anesthesiology, Second Affiliated Hospital, Zhejiang University School of Medicine, Hangzhou, China

**Keywords:** sleep disturbance, low back pain, Mendelian randomization, causal effect, insomnia

## Abstract

**Background:**

Previous observational studies have shown that low back pain (LBP) often coexists with sleep disturbances, however, the causal relationship remains unclear. In the present study, the causal relationship between sleep disturbances and LBP was investigated and the importance of sleep improvement in the comprehensive management of LBP was emphasized.

**Methods:**

Genetic variants were extracted as instrumental variables (IVs) from the genome-wide association study (GWAS) of insomnia, sleep duration, short sleep duration, long sleep duration, and daytime sleepiness. Information regarding genetic variants in LBP was selected from a GWAS dataset and included 13,178 cases and 164,682 controls. MR-Egger, weighted median, inverse-variance weighted (IVW), penalized weighted median, and maximum likelihood (ML) were applied to assess the causal effects. Cochran’s *Q* test and MR-Egger intercept were performed to estimate the heterogeneity and horizontal pleiotropy, respectively. Outliers were identified and eliminated based on MR-PRESSO analysis to reduce the effect of horizontal pleiotropy on the results. Removing each genetic variant using the leave-one-out analysis can help evaluate the stability of results. Finally, the reverse causal inference involving five sleep traits was implemented.

**Results:**

A causal relationship was observed between insomnia-LBP (OR = 1.954, 95% CI: 1.119–3.411), LBP-daytime sleepiness (OR = 1.011, 95% CI: 1.004–1.017), and LBP-insomnia (OR = 1.015, 95% CI: 1.004–1.026), however, the results of bidirectional MR analysis between other sleep traits and LBP were negative. The results of most heterogeneity tests were stable and specific evidence was not found to support the disturbance of horizontal multiplicity. Only one outlier was identified based on MR-PRESSO analysis.

**Conclusion:**

The main results of our research showed a potential bidirectional causal association of genetically predicted insomnia with LBP. Sleep improvement may be important in comprehensive management of LBP.

## Introduction

Low back pain (LBP) is a common disease and an important factor leading to limited activity, absenteeism, and disability (Woolf and Pfleger, 2003; Hartvigsen et al., 2018). In the National Health Interview Survey (NHIS), 31,044 participants were questioned and more than 25% of people stated they had experienced at least 1 day of LBP in the past 3 months (Deyo et al., 2006). A specific age limit does not reportedly exist for the occurrence of LBP. The incidence of LBP peaks in the third decade of life, and the prevalence increases until 60–65 years of age and then gradually declines (Golob and Wipf, 2014). Simultaneously, severe pain can reduce physical activity and may cause chronic musculoskeletal pain (Cimmino et al., 2011) which can significantly reduce the quality of life and social productivity of patients, resulting in high social costs. According to a rough estimate, the economic burden of LBP in Great Britain was close to 300 million pounds (Schofield et al., 2012). Unfortunately, the pathogenesis of LBP remains unclear and some scholars suggested it may be due to the interaction of biological, psychological, and social factors (Lall and Restrepo, 2017). Gender, obesity, aging, smoking, and mood disorders are several confirmed risk factors for LBP (Parreira et al., 2018; Shiri et al., 2019; Bento et al., 2020). Furthermore, sleep disturbances and the prognosis of patients with LBP has been an area of increased interest in recent years.

The potential association between sleep disturbances and LBP has been reported in many studies (O’Donoghue et al., 2009; van de Water et al., 2011; Murase et al., 2015). LBP was shown independently associated with short sleep duration and poor sleep quality (Murase et al., 2015). In another observational cohort study of 761 patients with LBP (Pakpour et al., 2018), after controlling for confounders such as depression, the researchers found that sleep disturbance was a risk factor for pain intensity which decreased after the sleep disorders were resolved. In addition, sleep condition and pain intensity were assessed in 80 patients with LBP. Generalized estimation equation (GEE) analysis showed that poor sleep quality and lower sleep efficiency may increase pain intensity the next day (Alsaadi et al., 2014). However, the above-mentioned observational studies did not yield a clear conclusion and unpredictable confounders might have produced reverse causality.

Mendelian randomization (MR) referred to a novel method which evaluate the causal association between a modifiable exposure and a clinically relevant outcome (Sekula et al., 2016). There were inherent defects in observational researches, and the presumption of the conclusions needs to strictly ensure the control of potential confounders. Considering that the alleles of genetic variants associated with exposure were randomly assigned, the performance of MR could effectively save time and economic costs, and help researchers explore the causal effect between exposures of interest and the outcome. Therefore, we conducted a bidirectional two-sample MR analysis to explore the causal relationship between the different traits of sleep disturbance and LBP.

In the present study, we hypothesized a causal relationship may exist between sleep disturbances and LBP. Although LBP has been primarily treated with analgesics, the application of sleep regulators may have potential usefulness in the comprehensive management of LBP.

## Materials and methods

### Data sources

In order to comprehensively assess the causal relationship between sleep disturbance and LBP, we selected five traits (insomnia, sleep duration, short sleep duration, long sleep duration, and daytime sleepiness) that can reflect sleep disturbance as genetic variants (Jia et al., 2022; Ni et al., 2022).

Insomnia is a common clinical condition characterized by difficulty initiating or maintaining sleep, accompanied by symptoms such as irritability or fatigue during wakefulness (Buysse, 2013). In the present study, the genetic variants of insomnia were obtained from the genome-wide association study (GWAS) among 336,965 individuals of European ancestry from the UK Biobank.

Other sleep traits such as sleep duration, short sleep duration, and long sleep duration, were described in detail in another GWAS summary statistics involving 128,266 subjects (Jones et al., 2016). Sleep duration was recorded based on self-reported sleep time and subjects who reported more than 18 h of sleep within 24 h were excluded from this study. Then, adjustment for age, gender, and study center was made to obtain the model residuals, and inverse normalizing was performed to ensure a normally distributed phenotype. Descriptions of short sleep duration and long sleep duration were defined based on average sleep duration. Short sleep duration refers people who slept less than 6 h per day on average and 28,980 subjects were included in this group. Long sleep duration reported an average of more than 9 h of sleep per day and 10,102 subjects were included in this group. GWAS summary statistics of daytime sleepiness were derived from the UK Biobank (Wang et al., 2019). A total of 452,071 participants of European genetic ancestry self-reported the frequency of daytime sleepiness using the question: ‘How likely are you to doze off or fall asleep during the daytime when you don’t mean to? (e.g., when working, reading or driving),’ with the answer categories ‘never’ (*N* = 347,285), ‘sometimes’ (*N* = 92,794), ‘often’ (*N* = 11,963), or ‘all of the time’ (*N* = 29). All participants who had taken any sleep-related medication were excluded. The latest GWAS summary statistics describing LBP (finn-b-M13_LOWBACKPAIN) was obtained by visiting the website^1^. This GWAS dataset consisting of 13,178 cases and 164,682 controls from European ancestry was identified in 2021.

### Selection of instrumental variables

Researchers screened the genetic variants that met the conditions based on strict quality control from the GWAS summary statistics of various sleep traits including insomnia, sleep duration, short sleep duration, long sleep duration, and daytime sleepiness. It was worth to emphasize that when performing MR analysis using genetic variants (usually single nucleotide polymorphisms [SNPs]) as instrumental variables (IVs), the IVs also need to satisfy three core assumptions: (1) Genetic variants are strongly associated with exposure factors; (2) Genetic variants are associated with the outcome only through the exposure of interest; and (3) Genetic variants are not associated with other confounders affecting the outcome (Boef et al., 2015). First, the single nucleotide polymorphisms (SNPs) associated with five sleep traits with genome-wide significance (*p* < 5e-8) were extracted. To obtain more IVs associated with the exposure of interest, relaxed thresholds, setting the maximum threshold to 5e-6, were used. This approach to threshold relaxation has been reported in other studies (Chen et al., 2020; Kwok and Schooling, 2021). Because the existence of linkage disequilibrium (LD) may cause corresponding bias, controlling LD before subsequent analysis was necessary. In this study, independent SNPs were selected by setting *r*^2^ < 0.001 and window size = 10,000 kb.

To further understand whether selected genetic variants were associated with potential confounders and the outcome, researchers visited PhenoScanner^2^, a website which provides details about genetic variants and phenotype information. We focused on physical and psychosocial factors including obesity, smoking, and mood disorders (e.g., anxiety and depression) associated with LBP. IVs that were significantly associated with the above confounders were eliminated before proceeding. However, because genetic variants were not readily available, this process was not performed under extremely stringent conditions.

### Mendelian randomization analysis

Statistical analyses were conducted using the R programming language (version 4.0.5). MR analysis was performed based on the “TwoSampleMR” package (version 0.5.6), and the “MRPRESSO” package (version 1.0) was used to apply MRPRESSO analysis to identified the outliers.

Various MR analysis approaches including MR-Egger, weighted median, inverse-variance weighted (IVW), penalized weighted median and maximum likelihood (ML) were performed to estimate the casual effects between sleep traits (insomnia, sleep duration, short sleep duration, long sleep duration, and daytime sleepiness) and LBP. The unbiased estimate of the causal effect can be obtained by IVW regression due to the non-existence of horizontal pleiotropy (Davies et al., 2019). The results of IVW analysis were considered as the major outcome. IVW was able to combine the effect of individual SNP on the outcome and obtained the ratio estimates (β). The ratio estimates were converted to acquire the corresponding odds ratios (ORs) and 95% confidence intervals (95%CIs). MR-Egger, which performing the directional pleiotropy, test of causal effect and the estimate of the causal effect, was an analytical method for MR using pooled genetic summary data (Burgess and Thompson, 2017). The pleiotropy of genetic variants may lead to the failure of the core assumption of IVs, but the causal effect can still be more accurately calculated when up to 50% of the information comes from invalid IVs in the method of weighted median estimator (Bowden et al., 2016). Both of the MR-Egger regression and weighted median could perform to improve the evaluation of IVW due to the robust estimates they could offer. Other methods such as ML (Hartwig et al., 2017) and penalized weighted median were mainly used to assess the robustness of MR results.

### Heterogeneity and horizontal pleiotropy

The horizontal pleiotropy of genetic variables is important because the results of MR analysis can be significantly affected and cause instability in the effect estimates. The test of correlation horizontal pleiotropy was mainly evaluated based on MR-Egger intercept and MR-PRESSO analysis; the former estimated the possibility of horizontal pleiotropy by calculating the term of intercept obtained after linear regression analysis. *F* statistics were calculated to evaluate the strength of the IVs. MR-PRESSO analysis identifies the outliers that may possess the characteristic of horizontal pleiotropy. The number of distributions in MR-PRESSO analysis was set to 1,000, and the robustness of MR analysis results was evaluated by comparing whether the casual relationship was affected before and after the outlier elimination. IVW and MR-Egger regression were applied to test the heterogeneity and Q statistics were calculated to quantitatively evaluate the heterogeneity. If the heterogeneity existed (*p* < 0.05), then the results of random effect IVW were dominant, otherwise it referred to the results of fixed effect IVW.

### Data visualization

To further evaluate whether there was a single SNP with a large contribution of pleiotropy that may cause deviation to the results, leave-one-out analysis was conducted to eliminate SNPs one by one and then re-estimate the causal effect. The forest plot was used to evaluate the effect estimation between the genetic variants and LBP, and MR-Egger regression and IVW to calculate the combined effects. If a correlation between parts of the SNPs and LBP was found, a specific rule determined whether a related SNP should be eliminated. More specifically, if *p*_exposure_ > *p*_outcome_, then the SNP was not included in the MR analysis. Funnel plot was used to evaluate the publication bias and applied to assess the potential directional pleiotropy in this study.

## Results

### Selection of instrumental variables

Supplementary Tables 1–3 shows the process of IV filtering in detail. SNPs were removed in the following situations. First, in the process of extracting SNPs from the outcome GWAS dataset, parts of the SNPs not found in the outcome dataset were removed. The results obtained from analysis of the four groups were as follows: insomnia-LBP (rs11804386), sleep duration-LBP (rs282086 and rs12537376), short sleep duration-LBP (rs573615914, rs546786239, rs8008258, and rs74500417), and long sleep duration-LBP (rs2387776, rs73196898, rs9915132, and rs4006399). Second, ambiguous SNPs with non-concordant alleles or palindromic SNPs with ambiguous strand were removed (Wu et al., 2020). In the analysis of insomnia-LBP, short sleep duration-LBP, long sleep duration-LBP, and daytime sleepiness-LBP, rs10280045, and rs2644128; rs9474974; rs2973993; rs3803763, rs58460356, rs61696052, rs6557066, rs72831782, and rs9475029, respectively, were eliminated. Third, genetic variants associated with the outcome and confounders were eliminated using PhenoScanner. Only rs73196898 was excluded in the MR analysis of long sleep duration and LBP in the present study.

### Mendelian randomization analysis

Different methods were used to evaluate the causal relationship between five sleep characteristics and LBP. To further investigate the effect estimation of LBP on sleep traits, reverse casual inference was also implemented, and Table 1 and Supplementary Table 4 show the MR analysis results in detail. Table 2 shows the details of GWAS summary statistics of LBP and sleep traits.

**TABLE 1:** Bidirectional MR analysis of casual effects between sleep traits and low back pain.

Exposure	Outcome	nSNP	IVW					MR-Egger						
			OR(beta)	95% CI	P value	Q statistics	P value	OR(beta)	95% CI	P value	Q statistics	P value	intercept	P value
Insomnia	Low back pain	27	1.954	1.119–3.411	0.0185	39.81181	0.0407019	6.269	1.388–28.313	0.025	36.01452	0.0713798	–0.0161066	0.1170137
Sleep duration	Low back pain	4	1.44	0.851–2.436	0.1745	2.340923	0.5047265	0.449	0.038–5.263	0.589	1.439539	0.4868645	0.0354446	0.4426188
Short sleep duration	Low back pain	10	1.45	0.575–3.656	0.4309	12.09611	0.2079454	3.071	0.303–31.08	0.370	11.35524	0.1823715	–0.0110508	0.4905873
Long sleep duration	Low back pain	19	1.566	0.887–2.765	0.122	28.88768	0.0497692	1.94	0.978–3.848	0.075	27.01214	0.0578901	–0.0088114	0.2924465
Daytime sleepiness	Low back pain	29	0.423	0.153–1.166	0.096	41.70184	0.0462439	0.116	0.0002–55.157	0.499	41.43407	0.0373934	0.0087731	0.6794595
Low back pain	Insomnia	20	1.015	1.004–1.026	0.006	25.77981	0.1364423	1.013	0.988–1.039	0.317	25.74813	0.105658	0.0001958	0.883349
Low back pain	Sleep duration	20	–0.017	–0.041–0.008	0.184	12.27915	0.873349	0.002	–0.046–0.05	0.925	11.47538	0.8731067	–0.0022579	0.3818029
Low back pain	Short sleep duration	20	1.004	0.993–1.016	0.443	12.61001	0.8579237	1.001	0.979–1.023	0.957	12.45992	0.822607	0.0004452	0.7029869
Low back pain	Long sleep duration	20	0.997	0.989–1.006	0.545	27.79357	0.0874826	0.998	0.978–1.019	0.870	27.77995	0.0654837	−0.0001024	0.9261895
Low back pain	Daytime sleepiness	20	1.011	1.004–1.017	0.001	20.70746	0.3531877	1.002	0.989–1.014	0.786	18.16353	0.4449299	0.0010332	0.1297471

**Exposure**	**Outcome**	**nSNP**	**Weighted median**				**Maximum likelihood**				**Penalized weighted median**			
			**OR(beta)**	**95% CI**		**P value**	**OR(beta)**	**95% CI**		**P value**	**OR(beta)**	**95% CI**		**P value**
Insomnia	Low back pain	27	2.095	1.024–4.286		0.043	1.998	1.262–3.166		0.003	1.958	0.947–4.046		0.070
Sleep duration	Low back pain	4	1.48	0.785–2.791		0.226	1.446	0.85–2.46		0.174	1.48	0.794–2.757		0.217
Short sleep duration	Low back pain	10	1.472	0.355–6.094		0.594	1.472	0.571–3.794		0.423	1.472	0.382–5.671		0.574
Long sleep duration	Low back pain	19	1.548	0.879–2.727		0.130	1.59	0.983–2.571		0.059	1.557	0.859–2.823		0.145
Daytime sleepiness	Low back pain	29	0.736	0.214–2.532		0.627	0.411	0.176–0.961		0.040	0.804	0.226–2.857		0.736
Low back pain	Insomnia	20	1.012	0.996–1.028		0.153	1.016	1.004–1.027		0.007	1.012	0.995–1.029		0.162
Low back pain	Sleep duration	20	–0.028	–0.063–0.007		0.122	–0.017	–0.042–0.008		0.184	–0.028	–0.061–0.005		0.101
Low back pain	Short sleep duration	20	1.006	0.99–1.022		0.451	1.006	0.991–1.022		0.446	1.006	0.991–1.022		0.446
Low back pain	Long sleep duration	20	0.997	0.985–1.01		0.694	0.997	0.988–1.006		0.520	0.996	0.983–1.009		0.532
Low back pain	Daytime sleepiness	20	1.005	0.996—1.015	0.261	1.011	1.004–1.017		0.001	1.005	0.996–1.015			0.250

nSNP, number of single nucleotide polymorphism; IVW, inverse-variance weighted; OR, odds ratio; 95% CI, 95% confidence interval.

**TABLE 2:** Description of six GWAS summary statistics.

Phenotype	Ancestry	Sample size	nSNP	Consortium	Data sources
Insomnia	European	336,965 participants	30	Neale Lab	ukb-a-13 (IEU OpenGWAS project)
Sleep duration	European	128,266 participants	6	UK Biobank	PMID: 27494321
Short sleep duration	European	28,980 cases and 81,204 controls	15	NA	PMID: 27494321
Long sleep duration	European	10,102 cases and 81,204 controls	24	NA	PMID: 27494321
Daytime sleepiness	European	104,786 cases and 347,285 controls	35	UK Biobank	PMID: 31409809
Low back pain	European	13,178 cases and 164,682 controls	20	FinnGen	finn-b-M13_LOWBACKPAIN (IEU OpenGWAS project)

nSNP, the number of single nucleotide polymorphism; PMID, ID of publication in the PubMed.

### Insomnia and low back pain

The causal effect of insomnia on LBP was the focus in the present study. The results showed a causal relationship between insomnia and LBP (IVW: OR = 1.954, 95% CI: 1.119–3.411, *p* = 0.019; MR-Egger: OR = 6.269, 95% CI: 1.388–28.313, *p* = 0.025; weighted median: OR = 2.095, 95% CI: 1.024–4.286, *p* = 0.043; maximum likelihood: OR = 1.998, 95% CI: 1.262–3.166, *p* = 0.003; penalized weighted median: OR = 1.958, 95% CI; 0.947–4.046, *p* = 0.070). Weak IVs due to the F statistics value was a low possibility (*F* = 13.928). The results of reverse causal inference indicated LBP can also have a causal effect on insomnia (IVW: OR = 1.015, 95% CI: 1.004–1.026, *p* = 0.006; MR-Egger: OR = 1.013, 95% CI: 0.988–1.039, *p* = 0.317; weighted median: OR = 1.012, 95% CI: 0.996–1.028, *p* = 0.153; maximum likelihood: OR = 1.016, 95% CI: 1.004–1.027, *p* = 0.007; penalized weighted median: OR = 1.012, 95% CI: 0.995–1.029, *p* = 0.162). The results of heterogeneity test are shown in Table 1 and Supplementary Table 5 and the output of heterogeneity analysis of insomnia-LBP indicated possible heterogeneity (MR-Egger: *Q* statistics = 36.01452, *p* = 0.07137978; IVW: *Q* statistics = 39.81181, *p* = 0.04070194). However, the reverse causal inference result appeared stable (MR Egger: *Q* statistics = 25.74813, *p* = 0.105658; IVW: *Q* statistics = 25.77981, *p* = 0.1364423). MR-Egger intercept was used to evaluate the horizontal pleiotropy and the results are shown in Table 1 and Supplementary Table 6 (intercept _(insomnia–LBP)_ = −0.01610656, *p*_(insomnia–LBP)_ = 0.11 70137; intercept_(LBP–insomnia)_ = −0.01610656, *p*_(LBP–insomnia)_ = 0.1170137). MR-PRESSO analysis showed no obvious outliers.

### Sleep duration and low back pain

A causal relationship between sleep duration and LBP was not found (IVW: OR = 1.44, 95% CI: 0.851–2.436, *p* = 0.175; MR-Egger: OR = 0.449, 95% CI: 0.038–5.263, *p* = 0.589; weighted median: OR = 1.48, 95% CI: 0.785–2.791, *p* = 0.226; maximum likelihood: OR = 1.446, 95% CI: 0.85–2.46, *p* = 0.174; penalized weighted median: OR = 1.48, 95% CI: 0.794–2.757, *p* = 0.217). A similar result was observed in reverse MR analysis (Supplementary Table 4). Abnormality was not found in the MR-PRESSO analysis, heterogeneity test, or MR-Egger intercept (Supplementary Tables 5–7).

### Short sleep duration and low back pain

The IVW analysis showed that genetic predisposition to short sleep duration did not increase the risk of LBP (OR = 1.45, 95% CI: 0.575–3.656, *p* = 0.431) and a similar output was found in reverse MR analysis (OR = 1.004, 95% CI: 0.993–1.016, *p* = 0.443). Heterogeneity test results and MR-PRESSO analysis in bidirectional causal inference were stable (Supplementary Tables 5, 7) and terms of intercept were −0.01105083 and 0.0004451583, respectively (Supplementary Table 6).

### Long sleep duration and low back pain

When estimating the causal effect of long sleep duration on LBP, results indicated the heterogeneity cannot be ignored (MR-Egger: *Q* statistics = 27.01214, *p* = 0.05789008; IVW: *Q* statistics = 28.88768, *p* = 0.04976916; Supplementary Table 5). Therefore, the output of random effect IVW analysis was considered to be more reliable. Next, the genetic predisposition to long sleep duration was shown to not affect the risk of LBP (OR = 1.566, 95% CI: 0.887–2.765, *p* = 0.122) and the results of reverse causal inference were similar (OR = 0.997, 95% CI: 0.989–1.006, *p* = 0.545) with terms of intercept −0.008811378 and −0.000102443, respectively (Supplementary Table 6). MR-PRESSO analysis showed no significant outliers (Supplementary Table 7).

### Daytime sleepiness and low back pain

The results of the sensitivity analysis are summarized in Supplementary Tables 5, 6; both MR-Egger and IVW analysis showed heterogeneity (MR-Egger: *Q* statistics = 41.43407, *p* = 0.03739338; IVW: *Q* statistics = 41.70184, *p* = 0.04624392) and random effect IVW analysis showed no causal relationship between daytime sleepiness and LBP. However, the MR-PRESSO analysis identified an outlier (rs4765936). When comparing the causal effect before and after removing this outlier, bias was not found in the adjusted result (raw: causal estimate = −0.3669115, *SE* = 0.493133, *T*-stat = −0.7440417, *p* = 0.4619627; outlier-corrected: causal estimate = −0.1740137, *SE* = 0.4572936, *T*-stat = −0.3805295, *p* = 0.705991; Supplementary Table 7). Reverse causal inference indicated that genetic predisposition to LBP could affect the risk of daytime sleepiness (IVW: OR = 1.011, 95% CI: 1.004–1.017, *p* = 0.00114; maximum likelihood: OR = 1.011, 95% CI: 1.004–1.017, *p* = 0.00144). MR-Egger intercept did not show horizontal pleiotropy [Intercept _(daytime sleepiness–LBP)_ = 0.008773135; Intercept _(LBP–daytime sleepiness)_ = 0.001033245]. Outliers were not identified in MR-PRESSO analysis and more detailed results are shown in Supplementary Tables 5–7.

### Visualization

**FIGURE 1:**
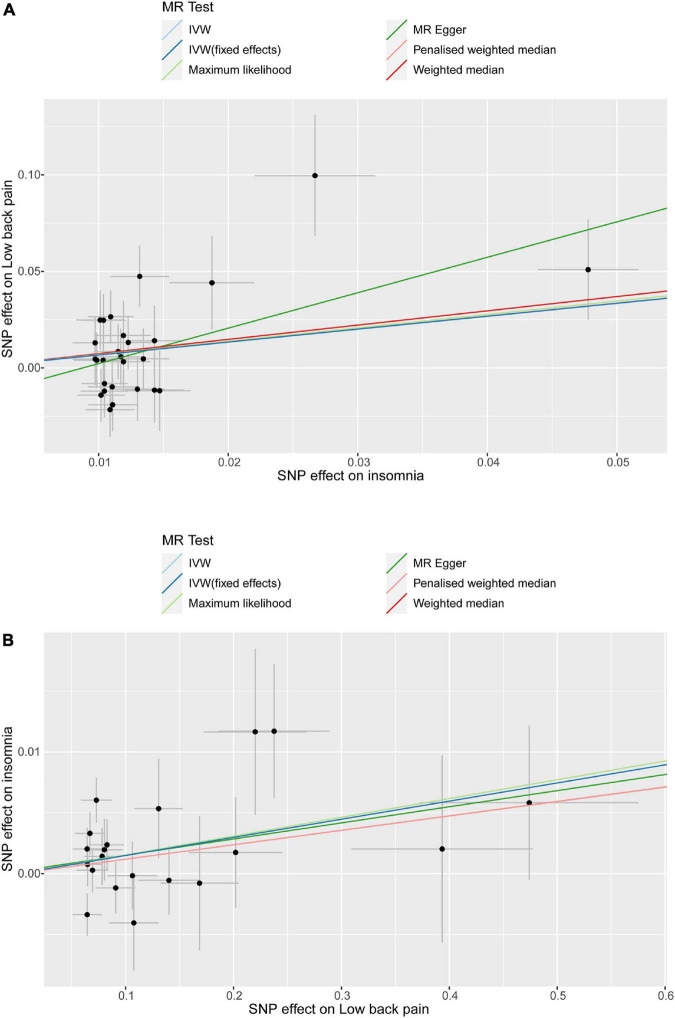
Individual estimates about the potential causal effect of insomnia and low back pain. The *x*-axis shows that the single nucleotide polymorphism (SNP) effect on insomnia in **(A)** and the *y*-axis shows the SNP effect on low back pain; **(B)** shows the results of the reverse causal inference. Several methods of MR analysis including MR Egger, weighted median, inverse variance weighted (IVW), maximum likelihood(ML) and penalized weighted median were performed.

**FIGURE 2:**
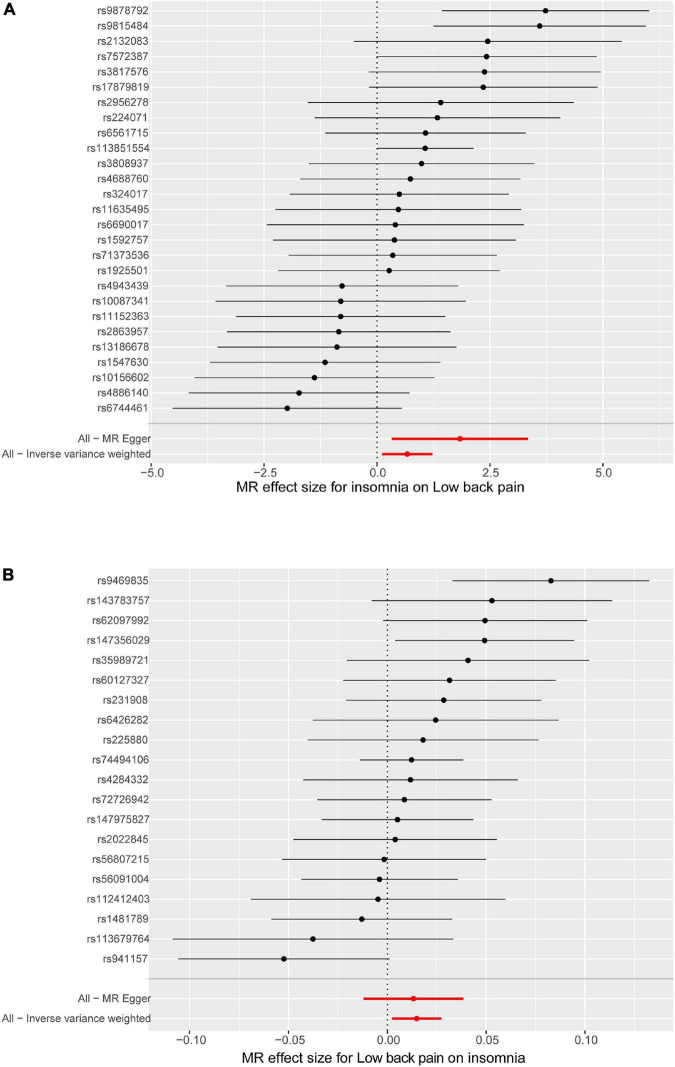
Forest plot of the causal effects between sleep disturbance and low back pain. In **(A)**, the *x*-axis shows that the MR effect size for insomnia on low back pain, and the *y*-axis shows the effect for each of the single nucleotide polymorphisms (SNPs); **(B)** shows the results of the reverse causal inference. MR Egger and inverse variance weighted (IVW) analyze the total effect of the genetic variants (or exposure) on the outcome.

**FIGURE 3:**
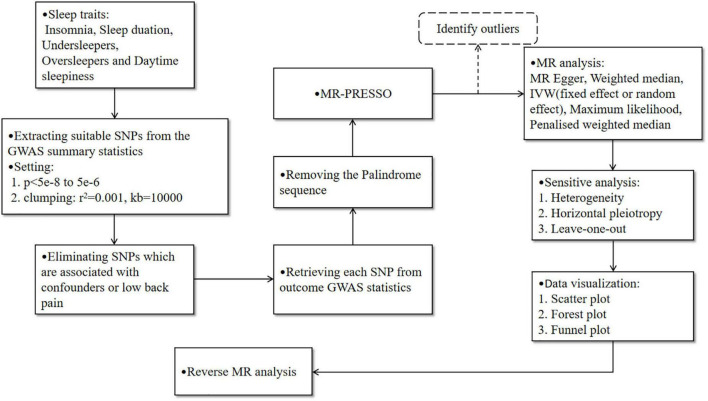
The design flow chart for the Mendelian randomization (MR) study. The dotted line indicates that the outliers may be eliminated after identification by MR-PRESSO, which needs to be judged based on the stability of the results before and after adjustment.

Leave-one-out analysis and funnel plot results are shown in Supplementary Figures 1, 2. Due to the existence of individual, potentially influential SNP, the results should be interpreted with caution. The funnel plot showed no probable directional pleiotropy. Figure 1 shows the individual putative causal effect between insomnia and LBP. The intercepts calculated in every method of MR analysis were close to zero which indicated the possibility of horizontal pleiotropy was low. A significant positive correlation was observed between insomnia and LBP. Figure 2 shows the causal effect estimates between each SNP and the outcome, and the combination of the effect estimates based on IVW and MR-Egger regression. Supplementary Table 3 shows the *p*_exposure_ of every SNP was less than *p*_outcome_, thus, additional SNPs were not removed in this procedure. Figure 3 shows the design flow chart for the MR study.

## Discussion

The study results supported our hypothesis. Specifically, MR analysis showed that insomnia significantly increases the risk of LBP (IVW: OR = 1.954, 95% CI: 1.119–3.411, *p* = 0.019). Based on the possible heterogeneity, the results were referred to the random effects IVW. Except the outcome assessed using penalized weighted median (OR = 1.958, 95% CI: 0.947–4.046, *p* = 0.070), the results of other MR analysis methods showed good consistency. All the ORs calculated using different methods were > 1, indicating the results of five MR analysis methods showed that insomnia patients increased the risk of LBP. The reverse MR analysis showed LBP also increased the risk of insomnia (IVW: OR = 1.015, 95% CI: 1.004–1.026, *p* = 0.006). Horizontal pleiotropy and outliers were not identified in sensitivity analysis, confirming the stability of results.

In previous observational studies, the potential association between sleep disturbance and LBP were reported. Axen and colleagues reported sleep disturbance in roughly 67% of the LBP patients included in a prospective study (Axen, 2016). This result reveals the possible coexistence between sleep disturbance and LBP. In a 3-year longitudinal study, a relationship was observed between sleep disturbance and LBP, and the duration and frequency of sleep disturbance were significantly associated with the development of LBP, becoming stronger as the two variables increased (Yabe et al., 2022). In addition, an interaction was found between LBP and sleep disturbance in which chronic daytime pain reduced the quality of nighttime sleep, subsequently causing worse pain the next day and further contributed to sleep disturbance (Kelly et al., 2011). Due to the growing amount of evidence from different research, strictly controlling for potential confounders is difficult, and a bidirectional association between sleep disturbance and LBP may exist (Finan et al., 2013; Alsaadi et al., 2014). Genetic variants and allelic randomization minimize the problem of confounding issues and reverse causation, providing stronger evidence than traditional observational studies in inferring cause-effect relationships (Davies et al., 2018).

The results of our two sample MR analysis supported the general theory of most observational research (Bahouq et al., 2013; Skarpsno et al., 2020). Sleep disturbances, such as insomnia, can significantly increase the risk of LBP, and the patients who experienced LBP were more likely to develop insomnia.

Previous MR studies have also showed the similar results. Sun et al. (2020) reported a causal relationship between sleep disturbance and chronic pain. Researchers replaced sleep disturbance with insomnia (the most common sleep disturbance) because they could not find GWAS summary statistics for sleep disturbance. Their study not only revealed the causal relationship between insomnia and chronic pain, but also provided the guidance for further research. In the present study, we limited the scope of the outcome, because LBP was the outcome that we were interested in. The inclusion of different sleep traits also helps us to explore the relationship between phenotypes other than insomnia and outcomes.

Debate remains regarding the specific mechanisms by which sleep deprivation affects pain. Nitric oxide (NO), considered a key element in the control of sleep and wake homeostasis, may have a significant role in both pain regulation and sleep. An increase of NO in rats with sleep deprivation was previously demonstrated, and that basal forebrain NO increases during sleep deprivation begin before frontal cortex increases in iNOS and NO. Other evidence showed application of a neuronal nitric oxide synthase (nNOS) inhibitor can attenuate mechanical hypersensitivity, indicating the increase of NO promotes hyperalgesia, and in the rat model of chronic pain, can worsen the pain inhibition activity in periaqueductal gray (PAG) area after sleep deprivation, resulting in severe pain (Kalinchuk et al., 2006; Smith et al., 2007; Tomim et al., 2016; Haack et al., 2020). Another study (Devine et al., 2019) reported that increased basal cortisol level and hyperreactivity of the Hypothalamus-pituitary-adrenal (HPA) axis to stressors were found in people who underwent insomnia. It was gratifying that this hyper-reactivity has been proved to involve in the relationship between insomnia and mechanical hypersensitivity (Goodin et al., 2012). Prostaglandin (PG), a classic inflammatory marker, was proved to mediate inflammatory pain. In an animal study, researchers found that the levels of PGs were significantly increased in the cerebrospinal fluid (CSF) of rats (Ram et al., 1997). Therefore, this potential mechanism may be complex and diverse.

On the other hand, we prefer that the results of MR analysis can provide guidance for the clinical treatment of LBP. For patients with chronic LBP, pain management and enhanced quality of life remains a significant and meaningful issue. Taking analgesic medications is an important method to manage chronic LBP (Koes et al., 2010; Shaheed et al., 2016), although frequent use to alleviate symptoms has less desirable therapeutic effects. Some evidence has shown that if LBP persists for longer than 12 weeks, physicians should concentrate more on pain management and enhancing quality of life rather than focusing on pain resolution (Golob and Wipf, 2014). For patients with chronic pain, sleep disturbance is an important factor that can reduce the quality of life.

Recently, the treatment of chronic pain was suggested to be impaired by poor and untimely intervention for insomnia (Nijs et al., 2018). Conversely, adding sleep regulator actively to pain-targeted therapy for patients suffering from insomnia may produce positive and unexpected effects. This assumption was supported by other research. In a double-blind trial, the researchers found significant improvement in sleep quality and pain grade in the observation group after adding eszopiclone to a standard naproxen pain relief regimen (Goforth et al., 2014). However, the long-term use of benzodiazepines should be taken into consideration due to the numerous adverse effects.

Melatonin (MT), a neurohormone that is mainly synthesized and secreted within the pineal gland, plays a significant role in humans as a powerful circadian regulator (Pfeffer et al., 2018). Externally applied melatonin can be used to treat the desynchronization of circadian rhythms that coursed by various factors. As a classic drug for the treatment of sleep disorders, melatonin has been used for improving sleep in patients with insomnia mainly because it does not cause hangover or show any addictive potential (Cardinali et al., 2012). In another study, researchers compared the efficacy in the observation group (3 mg melatonin 30–40 min before bedtime) and the control group (without melatonin) in patients with LBP for at least 12 weeks (Kurganova and Danilov, 2015). A significant reduction in pain intensity upon movement and in the resting state was observed in the observation group compared with the control group, which is in agreement with our MR results. Therefore, based on the above two studies, we speculated that the combination of sleep regulators and pain-directed therapy in integrated pain management of LBP patients may produce positive outcomes. However, the potential mechanism still needs more research to confirm.

As mentioned above, the results of MR analysis indicate a bidirectional causal relationship exists between insomnia and LBP. The treatment of LBP requires diversified and integrated management objectives, thus, other pain-related issues also should be considered in the treatment objectives for comprehensive management. Improvement of sleep disturbance is beneficial for improving the quality of life in patients with LBP, and use of sleep regulators may aid in achieving the goal of comprehensive pain management. But this hypothesis needs to be supported by more high-quality cohort studies with large samples or randomized controlled trials (RCTs).

## Limitations

The present study had several limitations. First, the participants included in this study were of European ancestry, thus, the results may not be equally applicable to other ethnic groups with different cultural backgrounds, geographical environments, living habits, and other factors. Second, several methods were used to identify and evaluate abnormal genetic variants, however, the possible effect of intermediation or pleiotropy on the results cannot be completely excluded. Then, sleep disturbance captures a much wider range of sleep concerns than just these characteristics, so more sleep traits may be considered in the MR analysis. Finally, it may be better to calculate the estimations within the subgroup based on gender stratification.

## Conclusion

A causal relationship was found between insomnia and LBP, and sensitivity analysis results tended to be robust. Sleep regulators may need to be considered in the comprehensive management of LBP with more evidences of perspective studies.

## Data availability statement

All the data used in this study came from the publicly available datasets. Related raw data can be visited and obtained in https://www.kp4cd.org/dataset_downloads/sleep and https://gwas.mrcieu.ac.uk/datasets/.

## Ethics statement

This study used the published articles or publicly available GWAS summary data. We did not collect additional raw data, and therefore approval from medical ethical committee is not required. Each study included has been approved by their institutional ethics review committees.

## Author contributions

GL, JT, TW, and YY collected and analyzed the data. YY and MY revised and reviewed the final approval of the manuscript. All authors revised and reviewed the manuscript.

## Footnotes

1
https://gwas.mrcieu.ac.uk/datasets/

2
http://www.phenoscanner.medschl.cam.ac.uk/

## Supplementary material

Supplementary Figure 1 Leave-one-out analysis.

Supplementary Figure 2 Funnel plot.

## Abbreviations

LBP: low back pain
CLBP: chronic low back pain
GEE: generalized estimation equation
GWAS: genome-wide association studies
SNP: single nucleotide polymorphism
IVs: instrumental variables
LD: linkage disequilibrium
IVW: inverse-variance weighted
MR: Mendelian randomization
ML: maximum likelihood
OR: odds ratio
95% CIs: 95% confidence intervals
NO: nitric oxide
nNOS: neuronal nitric oxide synthase
ESZ: eszopiclone
PAG: periaqueductal gray matter
MT: membrane receptors
NMDA: *N*-Methyl -D-aspartate.
