# Supplementary figures and images for: Causal association of sleep disturbances and low back pain: A bidirectional two-sample Mendelian randomization study
Source: Front Neurosci. 2022 Dec 2;16:1074605. doi: 10.3389/fnins.2022.1074605 (PMC9755499; doi:10.3389/fnins.2022.1074605)

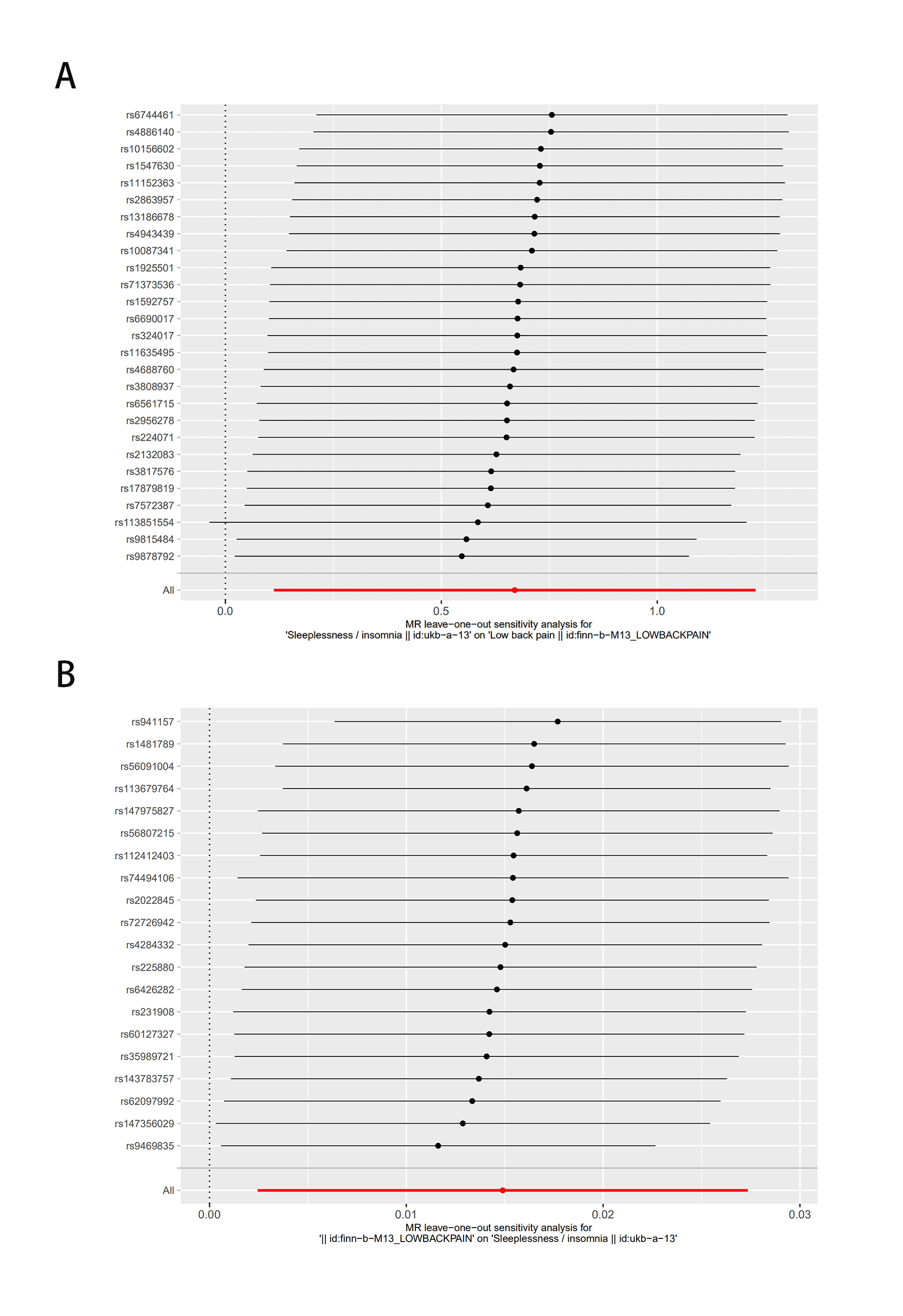

Supplement: Supplementary Figure 1 — Leave-one-out analysis. [file Image_1.TIF]

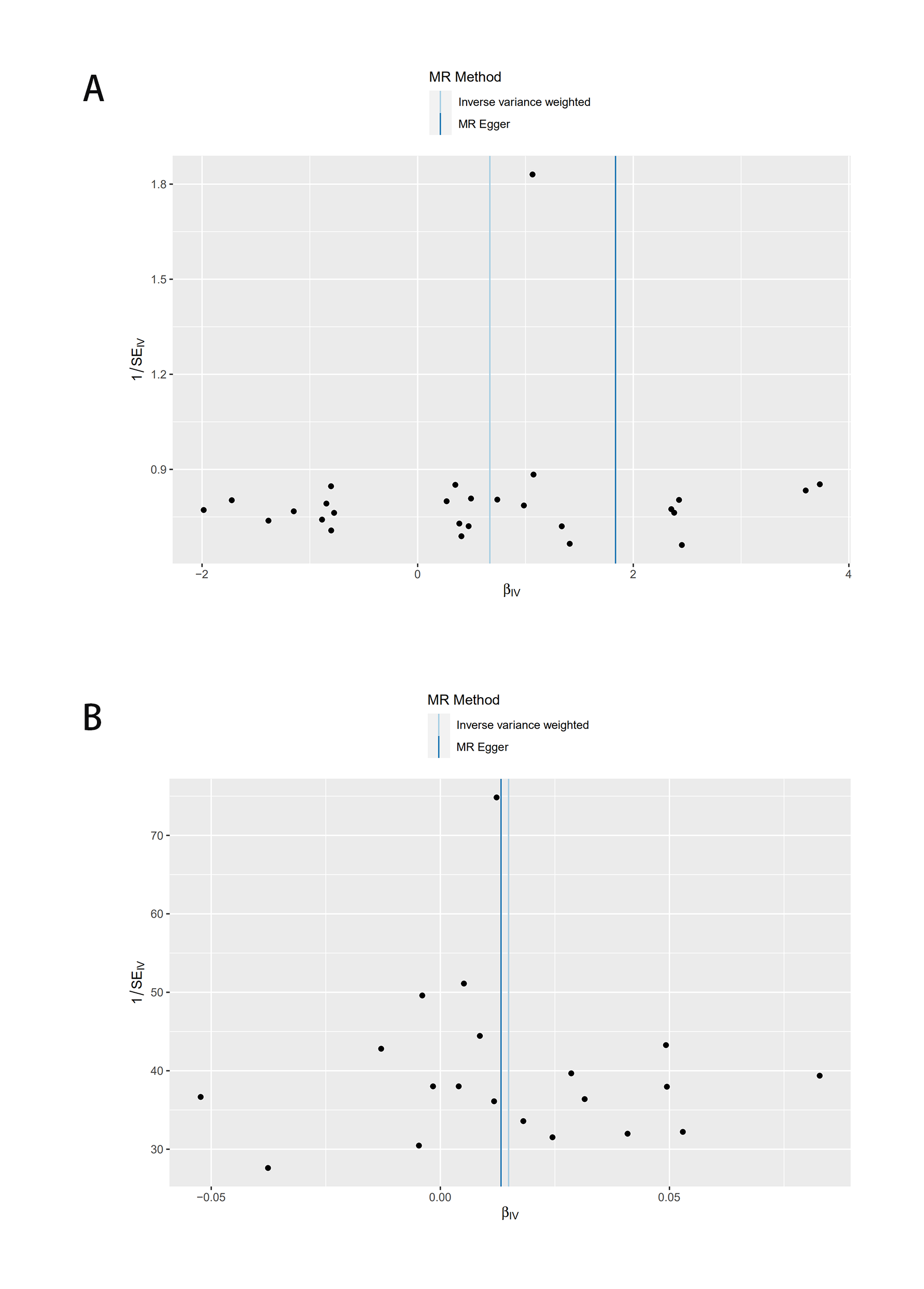

Supplement: Supplementary Figure 2 — Funnel plot. [file Image_2.TIF]
